# Belgian Case Series Identifies Non-Cow Mammalian Milk Allergy as a Rare, Severe, Selective, and Late-Onset Condition

**DOI:** 10.3390/nu17152393

**Published:** 2025-07-22

**Authors:** Sophie Verelst, Robbe Sinnesael, Firoz Taïbi, Sebastian Tuyls, Lieve Coorevits, Christine Breynaert, Dominique Bullens, Rik Schrijvers

**Affiliations:** 1 Allergy and Clinical Immunology Research Group, Department of Microbiology, Immunology and Transplantation, KU Leuven, 3000 Leuven, Belgium; robbe.sinnesael@kuleuven.be (R.S.); fayrouz1312@outlook.com (F.T.); sebastiaan.tuyls@zas.be (S.T.); lieve.coorevits@kuleuven.be (L.C.); christine.breynaert@kuleuven.be (C.B.); dominique.bullens@kuleuven.be (D.B.); rik.schrijvers@kuleuven.be (R.S.); 2 Clinical Division of Pediatrics, University Hospitals Leuven, 3000 Leuven, Belgium; 3 Clinical Division of General Internal Medicine, Allergy and Clinical Immunology, University Hospitals Leuven, 3000 Leuven, Belgium; 4 Clinical Division of Pediatrics, Jessa Hospital, 3500 Hasselt, Belgium

**Keywords:** mammalian milk allergy, buffalo milk allergy, cow’s milk allergy, mare milk allergy, sheep milk allergy, goat milk allergy, camel milk allergy, donkey milk allergy, anaphylaxis, cross reactivity, non-cow milk allergy

## Abstract

**Background**: Cow’s milk allergy (CMA) is the most common food allergy in children, typically resolving by adolescence. In contrast, the clinical spectrum of allergies to non-cow mammalian milk and their patterns of IgE cross-reactivity are less well documented. Nutritional differences between various mammalian milks may also impact dietary management in milk-allergic patients. **Objectives:** To characterize clinical features, onset age, and IgE cross-reactivity patterns of non-cow mammalian milk allergies in adult patients seen at a tertiary allergy center, and to compare these findings with published cases. **Methods:** A retrospective analysis of patients included in the “Extended Laboratory Investigation for Rare Causes of Anaphylaxis study” with mammalian milk allergy was performed using clinical history, skin testing, and serum-specific IgE measurements. Cross-reactivity patterns were assessed in selected cases using immunoblotting, specific IgE inhibition, and basophil activation testing, and compared with published reports of non-cow mammalian milk allergy. **Results**: In our case series of 22 patients with mammalian milk allergy and 10 healthy control subjects, 3 patients were identified with isolated adult-onset non-cow mammalian milk allergy (*n* = 1 buffalo milk; *n* = 2 mare milk), confirmed via immunoblotting and basophil activation testing. Streptavidin-based specific IgE measurement for buffalo cheese was positive in the buffalo milk allergic patient. The literature review identified 82 cases of non-cow mammalian milk allergy. These cases typically showed late onset (mean age 8.6 years; range 1–70 years), severe reactions (CoFAR (Consortium for Food Allergy Research) grade 3 or 4 in 66%, and one fatality), and selective sensitization (affecting sheep and/or goat, camel, mare, buffalo, donkey, or combinations thereof in 56, 10, 5, 5, 4, and 2 cases, respectively). **Conclusions**: Non-cow mammalian milk allergies are rare but generally present later in life with selective IgE cross-reactivity, differing from the broader cross-reactivity observed in CMA. This selectivity may allow for safe dietary alternatives. These findings underscore the need for improved diagnostics and personalized dietary management in this patient population.

## 1. Introduction

In recent decades, the prevalence of food allergies seems to be increasing, with cow’s milk (CM) emerging as one of the primary causative allergens in infants and young children [1,2]. Globally, cow’s milk allergy (CMA) affects 0.5% to 3% of children worldwide, accounting for up to 50 million children [2,3]. The natural tolerance induction in CMA typically occurs in approximately 65% of cases by the age of 12 years and in 80% by the age of 16 years [4]. However, 10–15% of CM-allergic children never become tolerant and can be termed ‘persistently cow’s milk allergic’ and require alternative milk sources [4,5]. In clinical practice, these patients often turn to plant-based milk analogues such as almond, soy, oat, and rice milk, which generally lack the allergenic proteins found in mammalian milks [6]. However, allergic reactions to certain plant-based milks, especially soy, have been reported in sensitized individuals. Additionally, the nutritional profile of plant-based milk substitutes is not always equivalent to that of cow’s milk; many lack sufficient levels of protein, fats, calcium, and essential vitamins unless adequately fortified. As an alternative, some patients consider milk from other mammalian species—such as goat, sheep, buffalo, camel, donkey, or mare. However, several of these mammalian milks are generally unsuitable for individuals with persistent CMA due to the high degree of IgE-mediated cross-reactivity and clinical symptoms [6,7]. A recent study by Katz et al. found that pediatric patients with CMA also tested positive for milk from deer, ibex, and buffalo [8]. Similarly, Sheehan et al. demonstrated that the majority of children with persistent CMA tested positive for water buffalo milk in SPTs [9]. However, there is a reported case of a 3-year-old boy with CMA who tolerated buffalo milk [10]. Prior studies also show that 92% of CMA patients react to goat milk, while only 4% react to mare milk and 8% to donkey milk [11,12,13]. Consequently, camel, donkey, and mare milk are considered suitable alternatives for CM-allergic patients, as both in vivo and in vitro studies indicate low levels of sequence similarity and minimal cross-reactivity with CM proteins [6,13,14,15]. From a nutritional standpoint, the exclusive use of donkey milk is not recommended due to its inadequate caloric content and low levels of essential nutrients such as iron and fats [16,17]. Mare milk is also considered nutritionally suboptimal, as it contains low levels of fats and calories—similar to donkey milk—although it does provide relatively higher concentrations of certain vitamins [6,16]. Finally, although camel milk could offer a nutritionally suitable alternative, its limited availability within Europe poses a significant challenge to its integration into newly developed hypoallergenic formulas [6,16].

Unlike CMA, primary allergies to non-cow mammalian milks are uncommon and rarely reported. Consequently, only a limited number of cases have been documented in the literature. However, the global consumption of mammalian milk products beyond traditional cow’s milk—both in dietary contexts and in cosmetic applications—is steadily increasing [16,18,19]. This trend raises the possibility of a corresponding rise in reported cases of allergies related to non-cow mammalian milk exposure in the future.

Non-cow mammalian milk allergy is characterized by an IgE-mediated hypersensitivity reaction to milk proteins derived from mammals other than cows, including goats, sheep, buffalo, camels, donkeys, and horses. These allergies are thought to arise from immune sensitization to specific epitopes on milk proteins that are unique to particular species and sufficiently distinct from CM proteins to elicit an allergic response [6]. The relative rarity of these allergies is likely explained by the high degree of similarity among milk proteins across mammalian species, which often leads to cross-reactivity. Clinically, symptoms of non-cow mammalian milk allergy closely resemble those of CMA and may include cutaneous manifestations, gastrointestinal disturbances, respiratory symptoms, and, in severe cases, anaphylaxis. The diagnostic evaluation of mammalian milk protein allergy involves a thorough clinical history, supplemented by skin prick testing and/or measurement of specific IgE antibodies to milk proteins. An oral food challenge remains the gold standard for confirming the diagnosis. However, establishing a diagnosis can be challenging, particularly in cases of non-cow mammalian milk allergy, due to the limited availability of commercial diagnostic tests and the lack of standardized extracts. With the increasing utilization of non-cow mammalian milk products worldwide, heightened awareness and improved understanding of these allergies are critical for accurate diagnosis and effective management.

Patients of this case series study were included in an ongoing observational prospective cohort study (s60734, starting in 2017) at the tertiary adult allergy center of University Hospitals Leuven, Belgium, of patients experiencing anaphylaxis, entitled “Extended Laboratory Investigation for Rare Causes of Anaphylaxis study” aiming to identify novel causes of anaphylaxis and/or improving molecular diagnosis for anaphylaxis. The impetus for this sub-investigation was the clinical identification of a patient with isolated buffalo milk allergy, which underscored limitations in available diagnostic tests and highlighted the need for further exploration of cross-reactivity. Additional cases of cow’s milk and non-cow’s milk allergy adult patients were identified (within study s60734, between 2017 and 2020). We hypothesized that non-cow mammalian milk allergy had a later onset compared to CMA and exhibited distinct patterns of IgE cross-reactivity. To test this, clinical features, age-of-onset, and IgE cross-reactivity patterns in non-cow mammalian milk allergy were evaluated. Among the 22 patients included, 3 rare cases of isolated, late-onset non-cow mammalian milk allergy were identified.

Our analysis prompted a broader retrospective analysis of previously included patients with CMA and other mammalian milk allergies within our cohort. To contextualize these findings, a comparison was made with published case reports of non-cow mammalian milk allergy from 1995 to 2024.

## 2. Materials and Methods

This case series analyzed adult patients with suspected IgE-mediated mammalian milk allergy, identified from the “Extended Laboratory Investigation for Rare Causes of Anaphylaxis” registry at the tertiary adult allergy clinic of University Hospitals Leuven, Belgium. This ongoing registry study, initiated in November 2017, collects pseudonymized clinical data and biobanked serum samples from patients presenting with clinical signs of anaphylaxis, regardless of etiology. Ethical approval was obtained from the Ethics Committee Research of University Hospitals KU Leuven (study number S60734).

### 2.1. Study Population and Design

For the current analysis, we retrospectively selected all adult patients (≥18 years) enrolled between November 2017 and 2020 whose anaphylactic episodes were suspected to be triggered by mammalian milk. Additional inclusion criteria included the ability to provide informed consent.

A total of 22 patients met the inclusion criteria and were categorized into three subgroups:

- 13 patients with confirmed CMA, including two with concomitant goat’s milk allergy;
- 6 patients sensitized to cow’s milk but clinically tolerant;
- 3 patients with selective non-cow mammalian milk allergy.

Additionally, 10 healthy non-allergic adults were included as negative controls.

### 2.2. Non-Cow Mammalian Milk Allergy Cases

This study focuses on three cases of isolated non-cow mammalian milk allergy:

- Patient 1 (AF129), a 48-year-old female with buffalo milk allergy;
- Patient 2 (AF220), a 31-year-old female with mare milk allergy;
- Patient 3 (HV206), a 62-year-old female with mare milk allergy.

Due to the absence of commercially available diagnostic tests for buffalo milk, additional testing was performed in Patient 1 (AF129). A streptavidin-based ImmunoCAP™ assay and immunoblotting were conducted on stored serum, while a basophil activation test was carried out prospectively using freshly drawn blood.

### 2.3. Skin Prick Test

Skin prick test (SPT) results were retrospectively retrieved from patient records where available. Tests had been performed using commercial extracts (ALK^®^ (Almere, The Netherlands), Hal Allergy^®^ (Leiden, The Netherlands), ALYOSTAL^®^ (Waterloo, Belgium)), supplemented by prick-to-prick tests with fresh extracts (e.g., buffalo meat and buffalo dairy products) prepared in the KU Leuven Allergy laboratory. Histamine dihydrochloride (1%) and saline (NaCl 0.9%) were used as positive and negative controls, respectively. A wheal size ≥ 3 mm was considered positive.

### 2.4. Total and Specific IgE Determination

Total and specific IgE values were either obtained from clinical records or measured retrospectively from stored serum samples using the ImmunoCAP™ Phadia 1000 system (Thermo Fisher Scientific, Gothenburg, Sweden). An IgE level ≥ 0.10 kU/L was considered positive. Tested allergens included whole cow’s milk, goat’s milk, mare’s milk, and individual cow’s milk proteins (α-lactalbumin, β-lactoglobulin, casein, and bovine serum albumin).

### 2.5. Preparation of Extracts

Fresh buffalo-derived samples, including buffalo milk, cheese, butter, yogurt, hamburger, and steak, were collected from the water buffalo farm ‘De Melkautomaat’ in St. Antonius, Zoersel, Belgium. The samples were homogenized, dissolved, and centrifuged at 3500–4500× *g*. The resulting supernatant was transferred to Eppendorf tubes and subjected to a second centrifugation at 21,000× *g* (4 °C for 30 min). The final supernatant was filtered through a 0.2 μm filter, aliquoted into tubes, and stored at −20 °C. The same procedure was applied to obtain protein extracts from various mammalian milks, including cow, mare, camel, yak, goat, and sheep milk. These extracts were subsequently used for immunological analyses to assess potential IgE reactivity and cross-allergenicity.

### 2.6. Sodium Dodecylsulfate-Polyacrylamide Gel Electrophoresis (SDS-PAGE)

SDS-PAGE was performed according to the ThermoFisher protocol. Invitrogen^TM^ NuPAGE^TM^ 10% Tris-Bis gel (NuPAGE, Invitrogen, Thermo Fisher Scientific, Waltham, MA, USA) was utilized to separate proteins based on their molecular weight. Samples containing 18 μg of allergen proteins were loaded onto the gel. Gel electrophoresis was conducted at 160 V for 50 min. The proteins were then visualized using the Pierce™ Silver Stain Kit (ThermoFisher, Waltham, MA, USA, Catalog Number 24612).

### 2.7. Immunoblotting

Immunoblotting was performed using stored serum samples to assess IgE-binding profiles and evaluate cross-reactivity. Proteins were electrophoretically transferred from the polyacrylamide gel to a polyvinylidene difluoride (PVDF) membrane using a transfer buffer (25 nM Tris, 192 mM glycine, 20% methanol) at 30 V for 1 h 20 min. The membrane was then blocked for 1 h at room temperature with PBST-5% HSA. Subsequently, it was incubated overnight at 4 °C with diluted patient serum (1:8 in PBST-1% HSA). Bound IgE was detected by incubating with monoclonal mouse anti-human IgE antibodies (clone XTE4, Genetex, Irvine, CA, USA) diluted 1:1000 in HSA-PBST for 1 h at room temperature. After several washing steps, the membrane was incubated with goat anti-mouse IgG antibody labeled with horseradish peroxidase (HRP) (p0447, Dako, Agilent, Santa Clara, CA, USA) diluted 1:20,000 in HSA-PBST for 2 h at room temperature. Following additional washes, bands were visualized using chemiluminescence detection with the Western Lightning Plus-ECL kit (PerkinElmer, Waltham, MA, USA). IgE binding was detected using ImageQuant LAS 500 (GE Healthcare Life Sciences, Marlborough, MA, USA), and protein bands were quantified by densitometry with ImageJ software (version 1.52; National Institutes of Health, Bethesda, MD, USA).

### 2.8. Basophil Activation Test (BAT)

A heparinized whole-blood sample from the patient was stimulated for 20 min at 37 °C in a water bath with allergens diluted in a basophil stimulation buffer (BSB) containing calcium and recombinant human interleukin-3 (rhIL-3, 60 ng/mL; PeproTech 200-03, Cranbury, NJ, USA). BSB served as a negative control, while polyclonal goat anti-human IgE (5 μg/mL; Sigma-Aldrich I6284, St. Louis, MO, USA) was used as an IgE-mediated positive control, and fMLP (50 ng/mL; Sigma-Aldrich F3506) functioned as an IgE-independent positive control [20]. The reaction was halted by incubation on ice for 5 min. Subsequently, cells were stained with phycoerythrin (PE)-conjugated anti-CD123 (20 ng/150 μL blood; BioLegend 306006, San Diego, CA, USA), fluorescein isothiocyanate (FITC)-conjugated anti-CD63 (80 ng/150 μL blood; BioLegend 353006), and Alexa Fluor 647-conjugated HLA-DR (20 ng/150 μL blood; BioLegend 307622) antibodies. Following erythrocyte lysis, cells were fixed with 1% paraformaldehyde (1% PFA). CD63 expression was then analyzed using the LSRFortessa flow cytometer (BD Biosciences, San Jose, CA, USA). Basophil activation was quantified as the percentage of CD63 +/CD123 +/HLA-DR- basophils. Data analysis was performed using FlowJo^®^ software (FlowJo LLC, version 10.4.2). A BAT is considered positive if at least two consecutive allergen concentrations each result in a CD63+ basophil response exceeding 10% above the negative control [21,22]. A BAT was prospectively conducted in Patient 1 (AF129), who exhibited isolated buffalo milk allergy, using freshly collected blood and various concentrations of both buffalo milk products and other mammalian milks. Unfortunately, for Patients 2 (AF220) and 3 (HV206), BATs could not be performed either at diagnosis or retrospectively, as valid execution of this test requires fresh blood samples, which were unavailable due to the retrospective nature of the study.

### 2.9. ImmunoCAP™ Assay with Biotinylated Buffalo’s Cheese Proteins

Since there are no commercially available kits for detecting specific IgE (sIgE) against buffalo milk, a diagnostic assay was developed using the Streptavidin ImmunoCAP™ system (Phadia, ThermoFisher Scientific, Uppsala, Sweden, Catalog number 14-5320-01). A pilot study using serum from patient 1 (AF129) revealed low sIgE antibody levels when buffalo milk proteins were biotinylated. The decision to biotinylate buffalo cheese proteins instead was based on the similarity in protein profiles between buffalo cheese and buffalo milk, as demonstrated by silver staining. The Streptavidin ImmunoCAP™ is a solid-phase system designed for the binding of biotinylated allergenic proteins, allowing for the detection of specific IgE. This assay is compatible with the UniCAP 100 Phadia system, enabling precise allergen-specific IgE measurements. The experimental approach in this study was based on the methodology described by Sander et al. [23]. Buffalo cheese proteins were biotinylated using the EZ-Link™ Sulfo-NHS-LC-Biotinylation Kit (Thermo Fisher, Catalog Number 21435) with a 20-fold molar excess per 1 mg/mL protein sample. The biotinylation reaction was carried out at pH 9 for 2 h at 4 °C. Free biotin was removed using Zeba Spin desalting columns (Thermo Fisher, Catalog Number 89891) or Vivaspin 6 sample concentrators (GE Healthcare, Catalog Number 28-9322-93). The biotinylated proteins were stored at −20 °C for up to 12 months. Fifty microliters of biotin-labeled buffalo cheese proteins were applied to pre-washed Streptavidin ImmunoCAPs™ (Phadia, ThermoFisher Scientific, Uppsala, Sweden, Catalog Number 14-5320-01) and incubated for 30 min at 37 °C. After incubation, the Streptavidin ImmunoCAPs™ were washed, and patient serum was added. Specific IgE levels were measured according to the UniCAP100 system protocol (Phadia, ThermoFisher Scientific, Uppsala, Sweden). To perform this ImmunoCAP™ assay, stored serum from the buffalo milk allergic patient (Patient 1, AF129) and from a non-allergic control subject (S149) was used. The highest levels of specific IgE antibodies were detected when 40 µg/mL of buffalo cheese proteins were applied.

### 2.10. ImmunoCAP™ Inhibition-Assay

The ImmunoCAP™ inhibition assay was performed on stored serum samples to assess cross- or co-sensitization. First, sIgE antibodies in patient sera were inhibited using CM proteins. A dilution series of CM proteins (100 μg/mL to 0.01 μg/mL) was prepared, and patient sera were incubated with these dilutions overnight at 4 °C. The next day, 50 μL of biotinylated buffalo cheese proteins were applied to Streptavidin ImmunoCAPs™, and the assay was conducted using the UniCAP100 system (Thermo Fisher Scientific/Phadia, Uppsala, Sweden). In a second setup, sIgE antibodies in stored serum were inhibited using buffalo milk proteins. A dilution series of buffalo milk proteins (100 μg/mL to 0.01 μg/mL) was prepared, and patient sera were incubated with these dilutions overnight at 4 °C. The following day, an ImmunoCAP™ RUN was performed using the UniCAP1000 system (Phadia AB, Uppsala, Sweden). The specific ImmunoCAPs™ tested included α-lactalbumin (f76, Catalog Number 14-4522-01), β-lactoglobulin (f77, Catalog Number 14-4523-01), and casein (f78, Catalog Number 14-4524-01).

### 2.11. Comprehensive Overview of the Literature on Non-Cow Mammalian Milk Allergy

To place our Belgian case series findings in context, a comprehensive comparison was made with previously published case reports on non-cow mammalian milk allergies. The following search terms were used in PubMed and Google Scholar to identify all case reports on mammalian milk allergy: mammalian milk allergy, buffalo milk allergy, cow’s milk allergy, mare milk allergy, sheep milk allergy, goat milk allergy, camel milk allergy, donkey milk allergy, anaphylaxis, and cross reactivity. Only case reports with an English abstract published between 1995 and 2024 were included. One abstract on buffalo milk allergy, presented at the EAACI Congress 2024, has been added. An additional manual search was conducted by screening the references of included articles, literature reviews, and related articles in PubMed and Google Scholar.

### 2.12. Statistical Analysis

The figures and statistical analysis were created using GraphPad Prism (GraphPad Software, version 10.1.1 for Windows, San Diego, CA, USA). Raw sIgE measurements were displayed on log-scale axes. Spearman correlation analysis was employed to examine the relationships between sIgE levels specific to buffalo cheese proteins and those for bovine milk, α-lactalbumin, β-lactoglobulin, and casein.

## 3. Results

### 3.1. Patient Characteristics and Allergy Testing

#### 3.1.1. Patient Characteristics

Table 1 summarizes the demographic data and clinical presentations for each of the 22 adult patients evaluated for suspected mammalian milk allergy, while a more detailed overview of the 3 patients diagnosed with non-cow mammalian milk allergy is provided in Table 2.

Of the 13 patients with CMA, 7 experienced symptom onset before the age of 2 years, while only 2 had an adult-onset CMA. In two patients, the age of onset was not documented in the clinical records. According to the Consortium of Food Allergy Research (CoFAR) grading, 7 out of 13 CM-allergic patients experienced grade 1 anaphylaxis, 1 out of 13 experienced grade 2, and 5 out of 13 experienced grade 3 anaphylaxis [24].

In the three patients with selective non-cow mammalian milk allergy, symptom onset occurred in adulthood in two out of three. Notably, only Patient 2 (AF220) reported a history of symptoms during childhood following the consumption of mare’s milk. These three patients presented with severe symptoms consistent with grade 3 anaphylaxis according to the CoFAR classification.

#### 3.1.2. Allergy Testing: Skin Prick Test, Total and Specific IgE Determination

Allergy testing results (SPT and total/specific IgE via CAP) for the 22 adult patients evaluated for mammalian milk allergy are summarized in Table 1.

SPT for CM was performed in 19 of the 22 patients. Among these, 5 patients had a negative result, 7 had an equivocal result, and 7 had a positive to strongly positive result. The prick-by-prick tests with buffalo milk and various buffalo cheeses were positive in patient 1 (AF129), while the test for mare milk was positive in patient 3 (HV206). No SPT for mare milk was performed in patient 2 (AF220). The CM SPT was negative in patients 1 (AF129) and 3 (HV206) but positive in patient 2 (AF220).

Specific IgE testing for CM and/or its components was performed in 21 of the 22 patients. Among these, only one patient had a completely negative result, namely patient 1 (AF129). Specific IgE for mare milk was positive in patient 2 (AF220) and patient 3 (HV206).

### 3.2. Allergen Identification in Non-Cow Mammalian Milk Allergy

**Figure 1:**
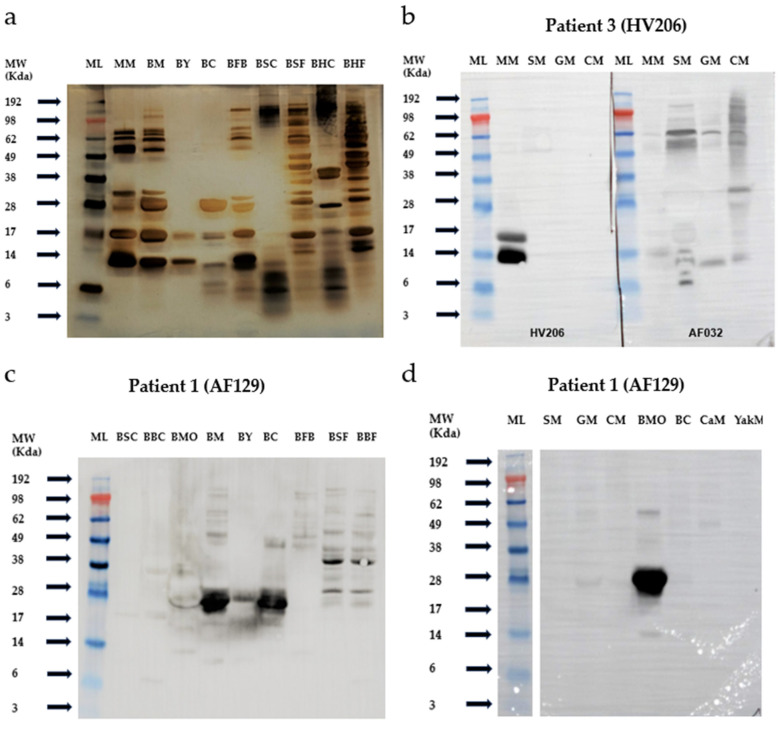
Patient reactivity to various mammalian milk sources and products as demonstrated by immunoblotting. (**a**) Silver-stained SDS-PAGE of reduced milk proteins (18 μg), separated by SDS-PAGE and visualized by silver staining to display the protein profiles. (**b**) Immunoblot analysis comparing IgE binding patterns in the isolated mare milk-allergic patient 3 (HV206) and a cow’s milk-allergic control patient (AF032). (**c**) Immunoblot of various buffalo milk-derived products in the isolated buffalo milk-allergic patient 1 (AF129). (**d**) Immunoblot of different mammalian milk sources tested in the same buffalo milk-allergic patient (AF129). Abbreviations: MW, molecular weight; ML, marker ladder (SeeBlue™); MM, mare milk; BM, buffalo milk; BY, buffalo yoghurt; BC, buffalo cheese; BFB, buffalo fresh butter; BSC, buffalo steak (cooked); BSF, buffalo steak (fresh); BBC, buffalo burger (cooked); BBF, buffalo burger (fresh); BMo, buffalo mozzarella; SM, sheep milk; GM, goat milk; CM, cow’s milk; CaM, camel milk; YakM, yak milk.

The results of the silver-stained SDS-PAGE are presented in Figure 1a. Protein bands with molecular weights of approximately 14 kDa and 17 kDa are likely to correspond to α-lactalbumin and β-lactoglobulin, respectively, derived from mare and buffalo milk [13,14]. The band observed between 19 kDa and 25 kDa is presumed to represent caseins originating from mare and buffalo milk, while the band detected at 66 kDa likely corresponds to serum albumin from both species [14]. Comparable protein profiles were observed across various buffalo-derived products, particularly within the 10–30 kDa molecular weight range.

### 3.3. Patient Reactivity

#### 3.3.1. Immunoblotting

The immunoblot of patient 3 (HV206) against native mare milk demonstrated a strong IgE-reactive band at molecular weights of 14 kDa and 17 kDa. Notably, there was no observed reactivity to sheep, goat, or cow’s milk (Figure 1b).

Patient 1 (AF129), with buffalo milk allergy, showed significant IgE reactivity against both buffalo cheese and milk (Figure 1c). However, no IgE binding was detected for other mammalian milks, including sheep, goat, cow, camel, and yak (Figure 1d). This indicates an isolated buffalo milk allergy without cross-reactivity to other milk sources. In comparison, control patient AF032, diagnosed with CMA, exhibited a degree of IgE cross-reactivity with sheep and goat milk (Figure 1b).

#### 3.3.2. BAT Reflecting Outcomes of Oral Food Provocation

**Figure 2:**
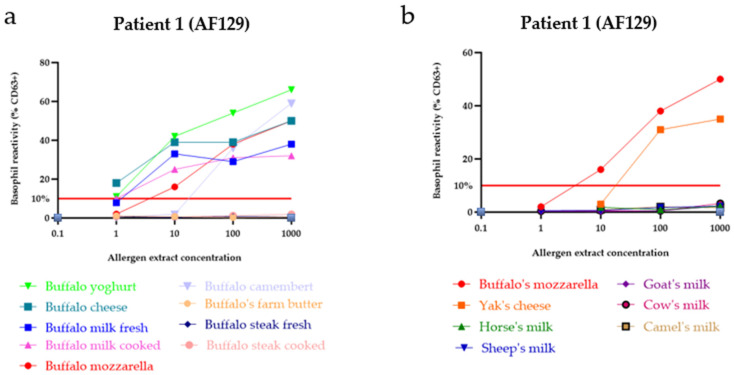
Patient reactivity of patient 1 (AF129) to various mammalian milk sources and products as demonstrated by BAT. Basophil reactivity is indicated by the proportion of CD63+ cells, measured via flow cytometry. (**a**) Dose–response curves of basophil activation upon stimulation with increasing concentrations of different buffalo milk products in patient 1 (AF129). (**b**) Dose–response curves of basophil activation with increasing concentrations of different mammalian milks in patient 1 (AF129), with CD63+ cells measured by flow cytometry.

Given the severity of the patients’ reactions, a provocation test with buffalo milk/cheese in patient 1 (AF129) and with mare milk in patients 2 (AF220) and 3 (HV206) was not conducted. BATs were developed to assess the potential introduction of certain mammalian milk-containing food products to the patients. In patient 1 (AF129), a BAT was performed with various buffalo-containing products as well as different mammalian milks. A positive BAT (upregulation of CD63+ basophils > 10%) was observed in response to buffalo mozzarella, buffalo cheese, buffalo camembert, buffalo milk (both raw and cooked), buffalo yogurt (Figure 2a), and yak cheese, although immunoblot with yak milk extract yielded negative results (Figure 1d). No upregulation of CD63+ basophils was observed with any other mammalian milks (Figure 2b). This finding supports the diagnosis of isolated buffalo milk allergy and suggests potential cross-reactivity with yak cheese. Of note, yak belongs to the same subfamily (Bovidae) as buffalo.

#### 3.3.3. Quantification of sIgE to Buffalo’s Cheese Proteins

**Figure 3:**
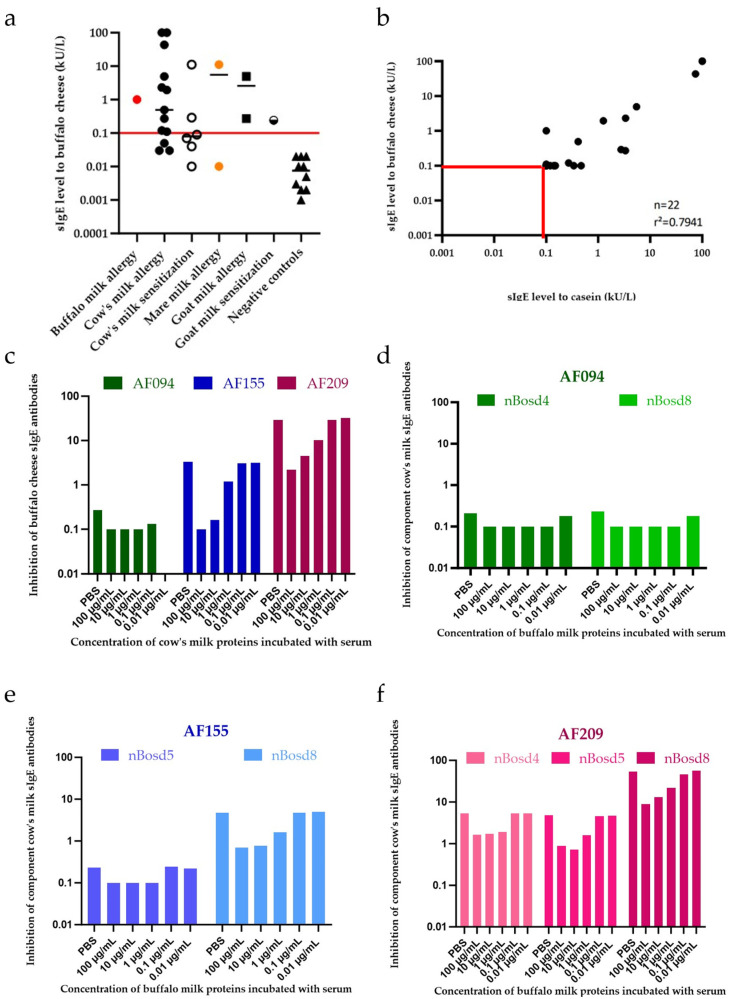
Cross-reactivity between mammalian milk sources assessed by specific IgE determination and inhibition assays. (**a**) Specific IgE (sIgE) detection using biotinylated buffalo cheese proteins. Closed circles/squares represent allergic patients; open or half-filled symbols indicate sensitized individuals; triangles represent negative controls. Red: patient 1 (AF129, isolated buffalo milk allergy); black: cow’s or goat’s milk allergic/sensitized patients; orange: patients 2 (AF220) and 3 (HV206), with isolated mare milk allergy. (**b**) Spearman correlation between sIgE levels to biotinylated buffalo cheese proteins and sIgE to cow’s milk casein. Sera were included from cow’s milk-allergic patients, sensitized but tolerant individuals, and patients with isolated buffalo or mare milk allergy. (**c**) ImmunoCAP™ inhibition assay showing inhibition of sIgE binding to buffalo antigens by increasing concentrations (100 to 0.01 µg/mL) of cow’s milk proteins in various patients. Raw data provided in Appendix A, Table A1. (**d**–**f**) ImmunoCAP™ inhibition assays showing inhibition of sIgE binding to bovine milk protein (f2) by buffalo milk proteins at increasing concentrations (100 to 0.01 µg/mL) in three individual cow’s milk-allergic patients: AF094 (**d**), AF155 (**e**), and AF209 (**f**). Raw data for each are available in Appendix A, Table A2.

Biotinylated buffalo cheese was coated on a streptavidin ImmunoCAP (Phadia, Thermofisher) and used to analyze sIgE to buffalo milk in a panel of 32 serum samples (Table 1). This included one patient with isolated buffalo milk allergy (patient 1, AF129); two patients with mare milk allergy (patient 2, AF220, and patient 3, HV206); thirteen patients with CM allergy (two of whom also had concomitant goat milk allergy); six CM-sensitized but tolerant patients; and ten non-allergic individuals. Patient 1 (AF129) was used as an internal positive control and demonstrated reactivity, whilst 10 non-allergic individuals showed no reactivity (Figure 3a). One patient with mare milk allergy (50%, patient 2, AF220), ten out of thirteen CM-allergic patients (77%), and two out of six CM-sensitized patients (43%) also showed reactivity.

### 3.4. Potential Cross-Reactivity of Proteins

#### 3.4.1. Cross-Reactivity Between Bovine Milk and Buffalo Cheese Proteins

Spearman’s correlation revealed a significant association between bovine milk protein (f2) and buffalo cheese proteins (r^2^ = 0.7426, *p* = 0.0001 ***). No significant correlation was found between sIgE to buffalo cheese proteins and α-lactalbumin (r^2^ = 0.2980; *p* = 0.1780). A weak but significant correlation was observed with β-lactoglobulin (r^2^ = 0.5510; *p* = 0.0079). Bovine casein sIgE levels correlated significantly with buffalo cheese sIgE levels (r^2^ = 0.7941, *p* < 0.0001 ****) (Figure 3b), suggesting potential cross-reactivity mostly via homologous casein proteins.

#### 3.4.2. Specific IgE Inhibition-Assay

Three CM-allergic donors with high (AF209), moderate (AF155), and low (AF094) sIgE titers to buffalo cheese were selected for the sIgE inhibition assay (Appendix A, Table A1 and Table A2). Figure 3c shows a dose-dependent inhibition of buffalo cheese-sIgE by pre-addition of CM proteins in all three patients. At the highest concentrations (100 and 10 µg/mL), CM proteins nearly completely inhibited IgE reactivity to buffalo cheese, whereas the lowest concentration (0.01 µg/mL) showed no inhibitory effect. As expected, a low dose of 0.1 µg/mL CM proteins only inhibited buffalo cheese-specific sIgE in patient AF094, who had the lowest sIgE titers to buffalo cheese. Conversely, high concentrations of buffalo milk proteins inhibited a substantial proportion of sIgE to bovine milk proteins in all three CM-allergic patients (Figure 3d–f). Patient AF155 showed no detectable sIgE to α-lactalbumin (nBos d 4), while patient AF094 had very low titers to β-lactoglobulin (nBos d 5). Patient AF209 exhibited the highest sIgE titers to all three CM components, with relatively elevated levels for casein (nBos d 8) compared to α-lactalbumin and β-lactoglobulin. Notably, buffalo milk proteins at high concentration (100 µg/mL) inhibited casein-sIgE (nBos d 8) by nearly 80%, further indicating cross-reactivity between these allergens.

### 3.5. Comprehensive Overview of the Literature on Non-Cow Mammalian Milk Allergy

**Figure 4:**
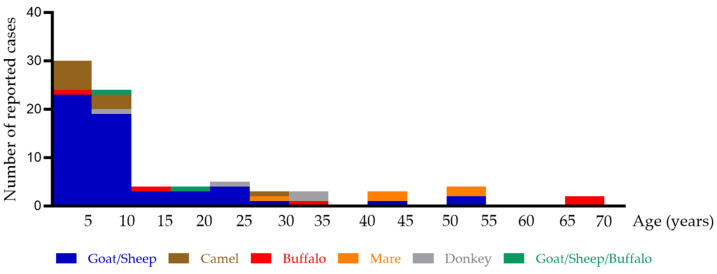
Comprehensive overview of reported cases of non-cow mammalian milk allergy in the literature. The number of reported allergy cases per milk type is shown by age group. A total of 82 cases of non-cow mammalian milk allergy have been reported to date. The distribution includes 5 cases of buffalo milk allergy (red), 4 cases of donkey milk allergy (grey), 5 cases of mare milk allergy (orange), 10 cases of camel milk allergy (brown), 56 cases of sheep and/or goat milk allergy (blue), and 2 cases involving both sheep/goat and buffalo milk allergy (green).

A literature search was conducted to contextualize our case series within the broader scientific literature on reported cases of non-cow mammalian milk allergy. A total of 82 cases of non-cow mammalian milk allergies have been reported since 1995, including 5 cases of buffalo milk allergy, 4 cases of donkey milk allergy, 5 cases of mare milk allergy, 10 cases of camel milk allergy, 56 cases of sheep and/or goat milk allergy, and 2 cases involving both sheep/goat and buffalo milk allergy (Table 3) [18,19,25,26,27,28,29,30,31,32,33,34,35,36,37,38,39,40,41,42,43,44,45,46,47,48,49,50,51,52,53,54,55,56,57,58,59]. Figure 4 provides a schematic representation of mammalian milk allergies reported in the literature, categorized by age group. Selective mammalian milk allergies appear to have a later onset, with a reported mean age of 8.6 years. The onset of donkey, mare, and buffalo milk allergies predominantly occurs in adulthood, whereas goat, sheep, and camel milk allergies tend to develop at a younger age. According to the literature, the youngest reported patient was one year old and experienced a reaction to camel milk [26], while the oldest was 70 years old and suffered an anaphylactic reaction to buffalo mozzarella [58]. A slight male predisposition was observed, with 50 out of 82 patients (61%) being male. A history of atopy was reported in 63 out of 82 patients (77%), and other food allergies were present in 27 out of 82 patients (33%). These different mammalian milk allergies often present with severe allergic reactions. Among the reported cases, 49 out of 82 patients (60%) experienced a CoFAR grade 3 reaction, while 5 out of 82 patients (6%) had a CoFAR grade 4 reaction, including one fatal case in an 8-year-old child due to a sheep milk allergy [40]. Additionally, 3 out of 82 patients reported anaphylaxis, but further details were not specified. Concomitant CMA was reported in only 6 out of 82 patients (7%), including 2 patients with a camel milk allergy and 4 patients with a sheep/goat milk allergy. These patients with concomitant CMA had an age range of 5 to 22 years.

## 4. Discussion

CMA remains the most prevalent form of milk allergy in both children and adults. Our 3 reported cases, along with 82 previously published cases, underscore the unusual nature of these selective allergies. Due to the high degree of amino acid sequence homology among mammalian milk proteins, cross-reactivity is common, and most individuals with CMA also react to milk from other mammals, such as goat, sheep, or buffalo [6]. However, selective mammalian milk allergies without concurrent CMA have only been described in 76 of the 82 published cases (Table 3). The three patients in our series tolerated CM and its derivatives without issue, despite having confirmed allergies to buffalo milk (Patient 1, AF129) and mare milk (Patient 2, AF220, and Patient 3, HV206).

Unlike CMA, which typically manifests within the first 6–12 months of life, selective mammalian milk allergies tend to develop later, with a mean onset age of 8.6 years, as reported in the literature. This observation aligns with our case series, in which all patients presented with adult-onset symptoms, except for Patient 2 (AF220), who had isolated mare’s milk allergy and reported mild allergic complaints during adolescence. However, definitive conclusions regarding the typical age of onset for non-cow mammalian milk allergy cannot be drawn, as the registry initially included only adults (≥18 years), with pediatric inclusion introduced at a later stage. CMA presents with a wide range of symptoms, from atopic dermatitis and gastrointestinal issues to systemic anaphylaxis, and remains a leading cause of severe allergic reactions in children—ranking third among food allergies and accounting for approximately 8–15% of anaphylactic episodes [6,16]. In contrast, selective mammalian milk allergies may be associated with an even higher frequency of severe reactions. Among reported cases, 60% involved a CoFAR grade 3 reaction and 6% a grade 4 reaction, including one fatality. All three patients with non-cow mammalian milk allergy in our series experienced CoFAR grade 3 reactions, consistent with the severity reported in the literature. However, the apparent predominance of severe cases may partly reflect publication bias, as milder presentations are likely underreported.

The diagnosis of IgE-mediated CMA typically relies on a consistent clinical history in combination with skin prick testing and/or sIgE measurement. While an oral food challenge remains the diagnostic gold standard, it is not always feasible—particularly in patients with a history of severe anaphylaxis. The challenges in diagnosing non-cow mammalian milk allergies have been frequently noted in several case reports, where the diagnostic work-up was often limited to skin prick testing, commercially available ImmunoCAP assays when accessible, and occasionally immunoblotting. To the best of the authors’ knowledge, the existing published cases of isolated buffalo milk allergy have not yet been thoroughly investigated or comprehensively characterized in the literature. Our case of a 48-year-old woman (patient 1 (AF129)) with isolated buffalo milk allergy further illustrates these diagnostic limitations. Prick-by-prick tests with buffalo milk and various buffalo cheeses yielded positive results. However, no commercial sIgE assays for buffalo milk proteins are available. Therefore, a streptavidin-based specific IgE assay (ImmunoCAP, Phadia) was developed in-house using biotinylated buffalo cheese proteins. Elevated sIgE levels were observed in patient 1 (AF129), as well as in 77% of CMA patients (10/13) and 43% of CM-sensitized patients (2/6), suggesting possible cross-reactivity or co-sensitization between buffalo and CM proteins. Given the severity of her reactions and the supporting diagnostic evidence, an oral food challenge was not performed. Instead, a BAT was conducted, providing functional confirmation of the allergy. This test yielded positive results, with >10% upregulation of CD63+ basophils in response to buffalo mozzarella, buffalo milk, buffalo yogurt, buffalo cheese, and buffalo camembert. In contrast, the test was negative for other mammalian milks, suggesting tolerance to these, in line with the clinical findings. Differentiating between cross-reactivity and true co-allergy remains challenging and is often based on empirical evidence. Inhibition assays demonstrated that the majority of CM-allergic patients with in vitro reactivity to buffalo milk were experiencing cross-reactivity. The diagnostic work-up of the buffalo milk allergy patient represents an initial step toward identifying the primary allergens responsible for this condition. Notably, buffalo casein emerged as a likely allergen, yet no cross-reactivity was observed with other mammalian milk products. In contrast, patients with current or prior CMA frequently exhibited cross-reactivity in the buffalo cheese sIgE assay, with the strongest correlation observed for CM casein. This suggests that highly species-specific epitopes may lead to sensitization in isolated cases, as observed in our case series of adult-onset patients, whereas recognition of less species-specific cross-reactive epitopes may contribute to allergy in our case series, which predominantly consists of childhood-onset cases. The mechanisms underlying this distinction are not yet fully understood, but it is possible that a disruption in pre-existing immune tolerance leads to highly specific allergies in later-onset cases. In contrast, the absence of prior tolerance may predispose individuals to a broader cross-reactivity in early-onset cases. Additionally, distinguishing cross-reactivity from co-allergy is often empirical and was addressed using inhibition tests.

In contrast, patients 2 (AF220) and 3 (HV206) did not undergo an extensive diagnostic workup, as commercially available sIgE assays for mare’s milk were available and sufficient to establish the diagnosis. Mare’s milk allergy is exceptionally rare, with only five cases reported in the literature to date, all presenting in adulthood. In most of these cases, sensitization was attributed to exposure through mare milk-based cosmetic products. Interestingly, some studies have proposed mare’s milk as a potential alternative for children with CMA, due to its relatively lower sequence homology to CM proteins [13,18,52]. However, mare’s milk proteins—particularly α-lactalbumin and β-lactoglobulin—have been implicated in rare IgE-mediated allergic reactions that occur independently of CMA. In patient 3 (HV206), immunoblotting against native mare’s milk revealed strong IgE reactivity at 14 and 17 kDa, corresponding to α-lactalbumin and β-lactoglobulin. These findings are consistent with immunoblot analyses reported in previously published cases [41,52,56]. Mare serum albumin (Equ c3) has been identified as a potential cross-reactive allergen between mare and CM [60], but in our patient 3 (HV206), no IgE binding to serum albumin was detected. Moreover, patient 3 (HV206) was able to tolerate CM, supporting the diagnosis of a selective mare’s milk allergy without cross-reactivity to CM. BATs were not feasible for these patients due to the lack of fresh samples. However, prior reports demonstrated positive BAT results for horse and donkey milk allergies, supporting BAT’s potential role in diagnosing selective mammalian milk allergies alongside conventional tests [41,52,56].

Our study has several limitations. First, the small number of cases limits the generalizability of our findings. Secondly, the possibility of publication bias should be considered, as severe cases are more likely to be reported, potentially overestimating the frequency of severe reactions. Thirdly, diagnostic workup was constrained by the limited availability of commercial assays for less common mammalian milks, impacting the ability to fully characterize allergic responses in all patients. Finally, the design with adult-only inclusion limits conclusions regarding pediatric onset and the natural history of these allergies. Despite these limitations, our findings contribute to a better understanding of selective mammalian milk allergies and highlight the need for further research and improved diagnostic tools to optimize patient care.

## 5. Conclusions

Primary allergies to non-cow mammalian milks are rare and tend to present later in life compared to CMA, which typically manifests in early childhood. While cross-reactivity between mammalian milks is common, our findings indicate that truly selective allergies are mediated by distinct protein allergens with limited overlap with cow’s milk proteins. This was demonstrated in our case series of three patients, all of whom tolerated cow’s milk but reacted to buffalo or mare milk. Our use of a streptavidin-based specific IgE ImmunoCAP™ assay highlights the feasibility of developing tailored in vitro diagnostics for non-standard food allergens. Altogether, isolated adult-onset non-cow mammalian milk allergies have been described. However, this work, combining a patient case series and literature study, demonstrates that non-cow mammalian milk allergy is rare, mostly severe, selective, and late-onset, contrasting with most CMA. Moreover, in vitro diagnostic tools are often lacking for these rare manifestations, as illustrated by our buffalo milk allergy case. These results emphasize the need for clinical awareness of such rare allergies and suggest a potential path toward improved diagnostic strategies.

## Figures and Tables

**Table 1:** Patient characteristics and allergy testing results of the 22 individuals included in this mammalian milk allergy case series.

Patient Characteristics					SPT				sIgE (kU/L)							Total IgE (kU/L)
Subject	Sex (M/F)	Age (Years)	Age at Onset (Years)	Clinical Presentation	Buffalo Milk	Cow`s Milk	Mare Milk	Goat Milk	Buffalo Cheese *	Cow`s Milk	n Bos d4	n Bos d5	n Bos d8	Mare Milk	Goat Milk	
AF209	M	22	0	Grade 3	NP	NP	NP	NP	43.2	64.2	5.63	4.85	74.2	NP	34	154
AF264	M	25	0	Grade 1	NP	2+	NP	NP	<0.10	0.99	0.29	<0.10	0.14	NP	NP	199
AF278	F	24	0	Grade 3	NP	4+	NP	NP	>100	>100	14.9	5.14	>100	NP	>100	663
AF094	F	25	1	Grade 1	NP	4+	NP	NP	0.49	0.51	0.25	0.11	0.41	NP	NP	1270
AF192	M	25	< 2	Grade 3	NP	2+	NP	NP	<0.10	0.19	<0.10	<0.10	0.12	NP	NP	35
AF168	F	27	2	Grade 1	NP	3+	NP	NP	0.11	1.01	0.47	0.14	<0.10	1.41	2.01	279
AF005	F	52	Child	Grade 1 + 3	NP	NP	NP	NP	0.27	3.3	0.31	0.59	3.34	NP	5.1	2565
AF220	F	31	Child	Grade 3	NP	4+	NP	NP	11.1	0.96	NP	NP	NP	8.97	NP	3536
AF155	M	26	8	Grade 1 + 3	NP	3+	NP	NP	4.92	30	<0.10	0.28	5.47	NP	NP	1388
AF259	M	33	9	Grade 1	NP	2+	NP	NP	2.31	2.38	0.53	0.11	3.35	NP	NP	1719
AF164	M	26	20	Grade 1	NP	3+	NP	NP	1.94	2.36	<0.10	1.49	1.26	NP	NP	748
AF268	F	51	36	Grade 3	NP	4+	NP	NP	>100	>100	0.21	6.51	>100	NP	NP	1196
AF129	F	48	48	Grade 3	3+	-	-	-	1	<0.10	<0.10	<0.10	<0.10	NP	<0.10	614
HV206	F	62	62	Grade 3	NP	-	3+	NP	<0.10	0.2	<0.10	0.29	<0.10	49.1	NP	330
AF092	M	60	NR	NR	NP	2+	NP	NP	<0.10	0.36	<0.10	<0.10	<0.10	NP	NP	141
AF097	F	22	NR	NR	NP	−	NP	NP	0.24	NP	NP	NP	NP	<0.10	0.52	>5000
AF100	F	31	NR	NR	NP	2+	NP	NP	<0.10	0.74	0.21	0.42	0.15	NP	NP	990
AF101	F	46	NR	NR	NP	2+	NP	NP	<0.10	0.82	<0.10	<0.10	<0.10	NP	NP	1393
AF112	M	51	NR	Grade 1	NP	2+	NP	NP	<0.10	7.81	1.09	0.87	0.47	NP	NP	>5000
AF182	F	21	NR	NR	NP	−	NP	NP	<0.10	0.42	NP	<0.10	0.34	NP	NP	853
AF210	F	66	NR	Grade 2	NP	1+	NP	NP	0.12	3.83	0.17	6.42	0.27	NP	NP	3490
AF249	M	26	NR	NR	NP	NP	NP	NP	0.29	2	<0.10	<0.10	2.75	NP	NP	0

The case series includes 13 patients with a confirmed CMA, 6 cow’s milk-sensitized patients, 1 patient with isolated buffalo milk allergy (patient 1 (AF129), indicated in red), and 2 patients with isolated mare milk allergy (patients 2 (AF220) and 3 (HV206), indicated in orange). Clinical data and skin prick test (SPT) results available at the time of inclusion were reviewed, together with total and specific IgE values obtained from previously measured or stored serum samples. In addition, an ImmunoCAP assay using biotinylated buffalo cheese was performed on stored serum samples to assess buffalo milk-specific IgE levels (*). Abbreviations: SPT, skin prick test; nBos d 4, α-lactalbumin; nBos d 5, β-lactoglobulin; nBos d 8, casein; NP, not performed; NR, not recorded.

**Table 2:** Clinical characteristics of the three patients with confirmed allergy to non-cow mammalian milks.

Patient	Patient Characteristics														CMA
	Sex	Age	Atopy				Presenting Symptoms							Culprit Food	
			AD	AA	ARC	FA	U	AE	GI	Resp	CVS	CNS	CoFar		
**1 (AF129)**	F	48 y	−	−	−	−	+	+	+	+	−	+	3	Buffalo ricotta	No
**2 (AF220)**	F	31 y	+	−	+	+	−	+	−	+	−	+	3	Mare milk	No
**3 (HV206)**	F	62 y	−	−	+	−	−	+	−	+	−	−	3	Mare milk/meat	No

Clinical characteristics are shown for one patient with isolated buffalo milk allergy (*patient 1* (*AF129*), indicated in red) and two patients with isolated mare milk allergy (*patients 2* (*AF220*) and *3* (*HV206*), indicated in orange). Abbreviations: AD, atopic dermatitis; AA, allergic asthma; ARC, allergic rhinoconjunctivitis; FA, pre-existing food allergy; U, urticaria; AE, angioedema; GI, gastrointestinal symptoms; Resp, respiratory symptoms; CVS, cardiovascular symptoms; CNS, central nervous system symptoms; CMA, cow’s milk allergy.

**Table 3:** Patient characteristics and allergy testing results from published case reports of non-cow mammalian milk allergy.

Reference	Patient Characteristics						Mammalian Milk Allergy											Diagnostic Test Non-Cow’s Milk			Diagnostic Test Cow’s Milk						CMA
	Sex	Age	Atopy				Type of MM	Presenting Symptoms									Culprit Food	SPT +	sIgE +	Immunoblot +	SPT +	sIgE +				Immunoblot +	
			AD	AA	ARC	FA		F	U	AE	GI	Resp	CVS	CNS	CoFar	Age					CM	CM f2	nBosd4	nBosd5	nBosd8		
Ehlayel et al. (2018) [26]	F	1 y	−	−	−	−	CaM	−	+	+	−	−	−	−	Grade 1	1 y	CaM	CaM	NP	NP	NP	NP	NP	NP	NP	NP	No
Ah-Leung et al. (2006) [59]	M	1.25 y	−	+	−	+	GM	−	−	−	−	+	−	−	Grade 3	1.25 y	GMC	GM/SM	GM/SM	NP	−	NP	NP	−	+	NP	No
Ehlayel et al. (2018) [26]	M	1.5 y	+	−	−	+	CaM	−	+	+		+	−	−	Grade 2	1.5 y	CaM	CaM	NP	NP	NP	NP	NP	NP	NP	NP	No
Ah-Leung et al. (2006) [59]	M	2 y	−	+	+	−	GM	−	+	−	−	+	−	−	Grade 3	2 y	GMC	GM/SM	GM/SM	NP	−	NP	NP	−	+	NP	No
Ah-Leung et al. (2006) [59]	F	2 y	+	+	−	+	GM	−	+	+	+	−	−	−	Grade 2	2 y	GMC	GM/SM	GM/SM	NP	−	NP	NP	−	−	NP	No
Martín et al. (2004) [28]	M	2 y	NM	NM	NM	NM	SM	−	+	+	−	−	−	−	Grade 1	2 y	SC	GM/SM	GM/SM	GM/SM	−	−	−	−	−	−	No
Umpiérrez et al. (1999) [29]	F	2 y	NM	NM	NM	+	GM/SM	−	−	+	−	+	−	−	Grade 3	2 y	GMC	GM/SM	GM/SM	GM/SM	−	−	−	−	−	+	No
Ibanez et al. (2006) [30]	F	2 y	NM	NM	NM	NM	GM/SM	NM	NM	NM	NM	NM	NM	NM	Anaph	2 y	SC	GM/SM	GM/SM	GM/SM	−	+	−	−	+	−	No
Khan et al. (2013) [31]	F	3 y	NM	NM	NM	NM	GM/SM	+	+	+	+	−	−	−	Grade 2	3 y	GMC	GM/SM	GM/SM	NP	−	−	NP	NP	NP	NP	No
Ehlayel et al. (2018) [26]	M	3 y	+	−	−	−	CaM	−	+	+	−	−	−	−	Grade 1	3 y	CaM	CaM	NP	NP	NP	NP	NP	NP	NP	NP	No
Ah-Leung et al. (2006) [59]	M	3 y	+	−	−	+	GM	−	+	+	−	+	−	−	Grade 3	3 y	GMC	GM	GM/SM	NP	−	NP	NP	−	+	NP	No
Ah-Leung et al. (2006) [59]	M	3 y	−	+	−	−	SM	−	−	−	−	+	−	−	Grade 3	3 y	SC	GM/SM	GM/SM	NP	−	NP	NP	−	−	NP	No
Ah-Leung et al. (2006) [59]	M	3 y	+	−	−	+	GM	−	−	+	−	+	−	−	Grade 3	3 y	GMC	GM	GM/SM	NP	−	NP	NP	−	−	NP	No
Ah-Leung et al. (2006) [59]	M	3 y	−	+	+	−	GM	−	−	+	−	+	−	−	Grade 3	3 y	GMC	GM/SM	GM/SM	NP	−	NP	NP	−	+	NP	No
Ah-Leung et al. (2006) [59]	M	4 y	+	+	−	+	SM	−	+	+	+	−	−	−	Grade 2	4 y	SC		GM/SM	NP	−	NP	NP	−	+	NP	No
Ah-Leung et al. (2006) [59]	M	4 y	−	+	+	+	SM	−	−	+	+	+	−	−	Grade 3	4 y	SC	GM/SM	GM/SM	NP	−	NP	NP	−	−	NP	No
Ah-Leung et al. (2006) [59]	M	4 y	+	+	+	−	SM	−	−	+	−	+	−	−	Grade 3	4 y	SMyoghurt/moussaka	GM/SM	GM/SM	NP	−	NP	NP	−	+	NP	No
De Boissieu et al. (2008) [32]	M	4 y	−	+	−	−	GM/SM	−	+	−	−	+	−	−	Grade 3	4 y	Feta	GM/SM	GM/SM	NP	−	NP	NP	NP	NP	NP	No
Mulé et al. (2020) [33]	M	4 y	+	+	NM	NM	GM/SM	+	+	−	+	+	−	+	Grade 3	4 y	Feta	GM/SM	NP	NP	−	NP	NP	NP	NP	NP	No
Piotin et al. (2023) [34]	F	4 y	+	+	+	+	BM	−	−	−	+	−	+	−	Grade 3	4 y	BMo	GM/SM/BM	GM/SM	NP	−	NP	−	+	+	NP	No
Martins et al. (2005) [35]	M	4 y	NM	NM	NM	+	GM/SM	−	+	+	−	−	−	−	Grade 1	2 y	GMC/SC	GM/SM	NP	GM	+	+	−	−	+	+	No
Ehlayel et al. (2018) [26]	F	4.5 y	−	−	−	−	CaM	NM	NM	NM	NM	NM	NM	NM	Grade 3	4.5 y	CaM	CaM	NP	NP	NP	NP	NP	NP	NP	NP	No
Calvani et al. (1998) [36]	M	5 y	+	+	+	+	SM	−	+	−	−	+	−	−	Grade 3	5 y	SC	GM/SM	NP	NP	−	−	NP	−	−	NP	No
Ehlayel et al. (2018) [26]	F	5 y	+	−	−	+	CaM	−	+	+	−	−	−	−	Grade 1	5 y	CaM	CaM	NP	NP	NP	NP	NP	NP	NP	NP	Yes
Ehlayel et al. (2018) [26]	M	5 y	+	−	−	−	CaM	NM	NM	NM	NM	NM	NM	NM	Grade 3	5 y	CaM	CaM	NP	NP	NP	NP	NP	NP	NP	NP	No
Pham et al. (2017) [18]	M	5 y	−	+	+	−	GM/SM	−	+	−	+	+	NM	NM	Grade 4	NM	GMC/SC	GM/SM	GM/SM	NP	+	+	NP	NP	NP	NP	No
Ah-Leung et al. (2006) [59]	M	5 y	+	+	−	−	SM	−	−	+	−	+	−	−	Grade 3	5 y	SC	GM/SM	GM/SM	NP	−	NP	NP	−	−	NP	No
Ah-Leung et al. (2006) [59]	F	5 y	−	+	−	−	GM/SM	−	+	−	−	+	−	−	Grade 3	5 y	SC/GM candy	GM/SM	GM/SM	NP	−	NP	NP	−	−	NP	No
Ah-Leung et al. (2006) [59]	F	5 y	−	+	−	+	GM	−	−	−	−	+	−	−	Grade 3	5 y	GMC	GM/SM	GM/SM	NP	−	NP	NP	−	−	NP	No
Ah-Leung et al. (2006) [59]	F	5 y	+	−	−	+	GM	−	+	+	−	−	−	−	Grade 2	5 y	GMC	GM	GM/SM	NP	−	NP	NP	−	−	NP	No
Ah-Leung et al. (2006) [59]	M	6 y	+	−	−	+	GM	−	−	−	+	−	−	−	Grade 1	6 y	GMC	GM/SM	GM/SM	NP	−	NP	NP	−	−	NP	No
Ah-Leung et al. (2006) [59]	M	6 y	+	+	+	+	GM	−	−	−	−	+	−	−	Grade 3	6 y	GMC/GM	GM/SM	GM/SM	NP	−	NP	NP	−	+	NP	No
Ah-Leung et al. (2006) [59]	M	6 y	+	+	−	+	GM	−	+	+	+	+	−	−	Grade 3	6 y	GMC	GM/SM	GM/SM	NP	−	NP	NP	−	+	NP	No
Ah-Leung et al. (2006) [59]	F	6 y	−	+	+	−	SM	−	+	−	+	+	−	−	Grade 3	6 y	SC	GM/SM	GM/SM	NP	−	NP	NP	−	+	NP	No
Vinas et al. (2014) [37]	M	6 y	−	−	−	+	GM/SM	NM	NM	NM	NM	NM	NM	NM	Grade 3	6 y	Roquefort	GM/SM	GM/SM	GM/SM	+	−	−	−	+	+	No
Mori et al. (2012) [38]	M	6 y	+	+	NM	−	GM/SM/BM	−	+	−	+	+	−	−	Grade 1–3	3/4/5y	Ewe/GMC/BC	GM/SM/BM	GM/SM	GM/SM	−	−	−	−	−	−	No
Al-Hammadi et (2010) [39]	B	6 y	+	+	NM	+	CaM	−	−	+	−	+	+	+	Grade 4	6 y	CaM	CaM	NP	NP	−	−	NP	NP	NP	NP	No
Ehlayel et al. (2018) [26]	F	6.5 y	+	−	−	+	CaM	NM	NM	NM	NM	NM	NM	NM	Grade 3	6.5 y	CaM	CaM	NP	NP	+	NP	NP	NP	NP	NP	Yes
Ah-Leung et al. (2006) [59]	M	7 y	−	+	+	+	GM	−	−	+	−	−	−	−	Grade 1	7 y	GMC	GM/SM	GM/SM	NP	−	NP	NP	−	+	NP	No
Ah-Leung et al. (2006) [59]	M	7 y	−	+	+	−	SM	−	−	−	+	−	−	−	Grade 1	7 y	SC	GM	GM/SM	NP	−	NP	NP	−	−	NP	No
Ah-Leung et al. (2006) [59]	M	7 y	+	+	−	−	SM	−	+	−	+	+	−	−	Grade 3	7 y	SM pasta	GM	GM/SM	NP	−	NP	NP	−	−	NP	No
Ah-Leung et al. (2006) [59]	M	7 y	−	+	−	−	GM	−	+	−	−	+	−	−	Grade 3	7 y	GM	GM	GM/SM	NP	−	NP	NP	−	+	NP	Yes
Ah-Leung et al. (2006) [59]	F	7 y	+	−	−	+	GM	−	−	+	−	−	−	−	Grade 1	7 y	GMC	GM	GM/SM	NP	−	NP	NP	+	−	NP	No
Vitte et al. (2008) [40]	M	8 y	+	NM	NM	NM	SM	NM	NM	NM	NM	+	+	+	Grade 4	8 y	CMC/SC	NP	GM/SM	NP	NP	+	NP	NP	NP	NP	No
Ehlayel et al. (2018) [26]	M	8 y	+	−	−	−	CaM	−	+	−	−	−	−	−	Grade 1	8 y	CaM	CaM	NP	NP	+	+	NP	NP	NP	NP	No
Vinas et al. (2014) [37]	M	9 y	−	−	−	+	GM/SM	NM	NM	NM	NM	NM	NM	NM	Grade 3	9 y	Cheese	GM/SM	GM/SM	GM/SM	−	+	−	−	−	+	No
Vinas et al. (2014) [37]	M	9 y	−	−	−	+	GM/SM	NM	NM	NM	NM	NM	NM	NM	Grade 4	9 y	CMC/GMC/SC	SM	GM/SM	SM	−	+	+	−	+	+	No
Martini et al. (2018) [41]	F	9 y	NM	NM	NM	+	DM	−	+	−	−	−	−	−	Grade 1	9 y	DMcream	DM	NP	DM/MM	−	NP	NP	NP	NP	−	No
Martins et al. (2005) [35]	M	9 y	NM	NM	NM	NM	GM/SM	−	−	+	−	+	−	−	Grade 3	6/7 y	Pizza cheese	GM/SM	NP	GM	−	−	NP	NP	NP	NP	No
Ah-Leung et al. (2006) [59]	F	9 y	+	−	−	−	GM	−	+	+	−	−	−	−	Grade 1	9 y	GMC	GM	GM/SM	NP	−	NP	NP	−	−	NP	No
Ah-Leung et al. (2006) [59]	M	10 y	+	−	+	+	GM/SM	−	+	−	−	−	−	−	Grade 1	10 y	Sandwich	GM	GM/SM	NP	−	NP	NP	−	−	NP	No
Ibanez et al. (2006) [30]	M	10 y	NM	NM	NM	NM	GM/SM	NM	NM	NM	NM	NM	NM	NM	Anaph	10 y	Cheese	GM/SM	GM/SM	GM/SM	−	+	−	−	+	+	No
Pazheri et al. (2014) [42]	M	10 y	NM	NM	NM	NM	SM	NM	NM	NM	NM	NM	NM	NM	Anaph	10 y	Romano/ricotta	NP	SM	NP	NP	−	NP	NP	NP	NP	No
Lamblin et al. (2001) [43]	M	10 y	+	−	−	−	GM/SM	−	+	+	−	+	−	−	Grade 3	10 y	SC	GM/SM	GM/SM	NP	+	+	NP	NP	+	NP	No
Dattou et al. (2005) [44]	F	11 y	+	+	NM	−	GM/SM/CM	+	+	+	−	+	−	−	Grade 1/3	11 y	CMC/GMC/SC	GM/SM	GM/SM	NP	+	+	NP	NP	NP	NP	Yes
Ah-Leung et al. (2006) [59]	M	12 y	−	−	−	−	GM	−	+	−	−	−	−	−	Grade 1	12 y	GMC	GM	−	NP	−	NP	NP	−	−	NP	No
Her et al. (2020) [45]	F	13 y	−	−	−	−	BM	−	−	+	+	+	−	+	Grade 3	13 y	BMo	BM	NP	NP	−	−	−	−	−	NP	No
Wüthrich et al. (1995) [25]	M	15 y	+	−	+	−	GM/SM	+	−	+	−	−	−	−	Grade 1	15 y	Feta	GM	GM/SM	NP	−	−	−	−	−	NP	No
Pétrus et al. (2011) [46]	M	16 y	NM	+	+	+	GM/SM/BM	−	+	+	−	+	−	−	Grade 3	11/16 y	CMC/GMC/SC	GM/SM/BM	GM/SM	NP	−	+	−	−	+	NP	No
Ah-Leung et al. (2006) [59]	F	16 y	−	−	+	−	SM	−	−	−	−	+	−	−	Grade 3	16 y	SC/SM yoghurt	GM/SM	GM/SM	NP	−	NP	NP	−	−	NP	No
Ah-Leung et al. (2006) [59]	M	16 y	−	−	+	−	GM	NM	NM	NM	NM	NM	NM	NM	Grade 3	16 y	GMC	GM/SM	GM/SM	NP	−	NP	NP	−	−	NP	No
Beaumesnil et al. (2013) [47]	M	20 y	+	NM	NM	−	GM	−	+	+	−	+	−	−	Grade 3	4/7 y	GMC	GM	GM	GM	+	+	+	+	+	+	Yes
Beaumesnil et al. (2013) [47]	M	22y	NM	NM	NM	−	GM/CM	NM	NM	NM	NM	NM	NM	NM	Grade 4	17 y	GMC/CMC	GM/SM	GM	GM	+	+	+	−	+	−	Yes
Lamblin et al. (2001) [43]	M	22 y	+	+	NM	NM	GM/SM	−	+	+	−	+	+	−	Grade 3	22 y	GMC	NP	GM/SM	NP	NP	−	−	−	NP	NP	No
Wüthrich et al. (1995) [25]	M	25 y	−	−	−	−	GM/SM	−	+	+	−	+	−	−	Grade 3	25 y	GMC/SC	GM	GM/SM	NP	−	−	−	−	−	NP	No
Peeters et al. (2017) [48]	F	25 y	+	+	+	−	DM	+	−	+	−	+	+	+	Grade 3	25 y	DM	DM	NP	NP	−	−	−	−	−	NP	No
Suzuki et al. (2021) [49]	F	25 y	+	+	NM	−	SM	−	−	+	+	+	−	−	Grade 3	21/22/23 y	Picorino	GM/SM	SM	SM	−	−	−	−	−	−	No
Tavares et al. (2007) [50]	F	27 y	NM	+	+	NM	GM	−	+	−	−	−	−	−	Grade 1	24 y	Caprine	GM	−	GM	−	−	−	−	−	−	No
Ehlayel et al. (2018) [26]	F	30 y	−	−	+	−	CaM	NM	NM	NM	NM	NM	NM	NM	Grade 3	30 y	CaM	CaM	NP	NP	NP	NP	NP	NP	NP	NP	No
Fanta et al. (1998) [19]	F	30 y	NM	+	NM	NM	MM	−	+	+	−	+	−	−	Grade 3	30 y	MM	MM	MM	MM	NP	−	NP	NP	NP	NP	No
Martini et al. (2018) [41]	F	33 y	+	−	+	−	DM	+	−	+	−	−	−	−	Grade 1	33 y	DM	DM	NP	DM/MM	−	NP	NP	NP	NP	−	No
Bahal et al. (2017) [51]	F	34 y	NM	NM	NM	NM	BM	−	−	+	−	−	−	−	Grade 1	NM	BMO	BM	−	NM	NM	−	NM	NM	NM	NM	No
Giorgis et (2018) [52]	F	35 y	NM	+	+	−	DM	−	−	−	−	+	−	−	Grade 3	35 y	DM	DM	−	DM	−	−	−	−	−	NP	No
Alvarez et al. (2002) [53]	M	43 y	−	−	−	−	SM	−	−	−	+	+	+	−	Grade 3	43 y	SM-curd	GM/SM	GM/SM	GM/SM	NP	−	−	−	−	−	No
Doyen et al. (2013) [54]	F	44 y	NM	NM	+	NM	MM	+	−	−	+	−	−	−	Grade 1	44 y	MM cosmetics/pills	GM/SM/MM	GM/SM/MM	MM	−	+	+	+	+	NP	No
Verhulst et al. (2016) [55]	F	45 y	NM	NM	NM	NM	MM	−	+	+	−	−	−	−	Grade 1	45 y	MM/CM cream	MM	MM	NP	−	+	+	−	−	NP	No
Gall et al. (1996) [27]	F	51 y	NM	NM	NM	NM	MM	−	−	+	−	+	+	−	Grade 3	51 y	MM granulate	MM	MM	MM	−	NP	−	−	−	NP	No
Lamblin et al. (2001) [43]	F	52 y	−	−	−	−	GM/SM	−	+	−	−	+	−	+	Grade 3	52 y	GMC/SC	GM/SM	GM/SM	NP	−	−	−	−	NP	NP	No
Robles et al. (2007) [56]	F	53 y	NM	+	+	NM	MM	+	−	−	−	+	−	−	Grade 3	53 y	MM	MM	MM	NP	NP	NP	NP	NP	NP	+	No
Vinas et al. (2014) [37]	M	54 y	−	−	−	−	GM/SM	NM	NM	NM	NM	NM	NM	NM	Grade 3	54 y	CMC/GMC/SC	GM/SM	SM	SM	+	−	−	−	−	+	No
Ruiz Del Barrio et al. (2024) [57]	M	67 y	NM	NM	+	NM	BM	−	−	+	−	+	−	+	Grade 3	67 y	BMo	BM	NP	BM	−	NP	−	−	−	NP	No
Broekaert et al. (2008) [58]	M	70 y	NM	NM	NM	−	BM	−	+	+	−	+	−	+	Grade 3	70 y	BMo	BM	NP	BM	−	−	−	−	−	−	No

Since 1995, a total of 82 cases have been reported. In addition to demographic information and clinical presentation, this table summarizes the results of diagnostic tests performed and indicates whether patients had a selective allergy to a specific mammalian milk or showed evidence of cross-reactivity. Abbreviations: AD, atopic dermatitis; AA, allergic asthma; ARC, allergic rhinoconjunctivitis; FA, food allergy; U, urticaria; AE, angioedema; GI, gastrointestinal symptoms; Resp, respiratory symptoms; CVS, cardiovascular symptoms; CNS, central nervous system symptoms; SPT, skin prick test; sIgE, specific IgE; CaM, camel milk; GM (C), goat milk (cheese); SM, sheep milk; MM, mare milk; DM, donkey milk; BM, buffalo milk; SC, sheep cheese; CMC, cow’s milk cheese; BC, buffalo cheese; Bmo, buffalo mozzarella; nBos d 4, α-lactalbumin; nBos d 5, β-lactoglobulin; nBos d 8, casein; CMA, cow’s milk allergy; NM, not mentioned; NP, not performed; “+”, positive/present; “−”, negative/absent.

## Data Availability

The raw data supporting the conclusions of this article will be made available by the authors on request.

## Abbreviations

The following abbreviations are used in this manuscript:

AA	allergic asthma
aIgE	anti-immunoglobuin E
AD	atopic dermatitis
ARC	allergic rhinoconjunctivitis
BAT	basophil activation test
BSB	basophil stimulation buffer
BM	buffalo milk
BC	buffalo cheese/buffalo camembert
BR	buffalo ricotta
Bmo	buffalo mozzarella
CaM	camel milk
CM	cow’s milk
CMA	cow’s milk allergy
CoFAR	Consortium for Food Allergy Research
DM	donkey milk
fMLP	n-formylmethione-leucyl-phenylalanine
GM	goat milk
IgE	immunoglobulin E
MM	mare milk
nbosd4	α-lactalbumin
nbosd5	β-lactoglobulin
nbosd6	bovine serum albumin
nbosd8	casein
OFC	oral food challenge
PBS	phosphate-buffered saline
kDa	kilodalton
rhIL-3	recombinant human interleukin-3
SDS-PAGE	sodium dodecylsulfate-polyacrylamide gel electrophoresis (SDS-PAGE)
SM	sheep milk
sIgE	specific immunoglobulin E
SPT	skin prick test

## Appendix A

**Table A1:** Raw data from the ImmunoCAP™ inhibition assay assessing the inhibition of specific IgE (sIgE) binding to buffalo milk antigens by pre-incubation with increasing concentrations of cow’s milk proteins (100 μg/mL to 0.01 μg/mL). Three CM-allergic donors with high (AF209, in red), moderate (AF155, in blue), and low (AF094, in green) sIgE titers to buffalo cheese were selected for the sIgE inhibition assay.

	Patient AF94	Patient AF155	Patient AF209
Concentration of CM in Serum	BM (kU/L)	BM (kU/L)	BM (kU/L)
PBS	0.27	3.28	29.1
100 µg/mL	<0.10	<0.10	2.22
10 µg/mL	<0.10	0.16	4.51
1 µg/mL	<0.10	1.19	10.1
0.1 µg/mL	0.13	3.11	29.5
0.01 µg/mL	ND	3.13	32.6

Abbreviations: CM, cow’s milk; BM, buffalo milk; PBS, phosphate-buffered saline; ND, not determined.

**Table A2:** Raw data from the ImmunoCAP™ inhibition assay evaluating the inhibition of specific IgE (sIgE) antibodies to f2 bovine milk protein by pre-incubation with increasing concentrations of buffalo milk proteins (100 μg/mL to 0.01 μg/mL). Three CM-allergic donors with high (AF209, in red), moderate (AF155, in blue), and low (AF094, in green) sIgE titers to buffalo cheese were selected for the sIgE inhibition assay.

	Patient AF94			Patient AF155			Patient AF209		
Concentrations of BM in Serum	nBosd4 (kU/L)	nBosd5 (kU/L)	nBosd8 (kU/L)	nBosd4 (kU/L)	nBosd5 (kU/L)	nBosd8 (kU/L)	nBosd4 (kU/L)	nBosd5 (kU/L)	nBosd8 (kU/L)
PBS	0.21	<0.10	0.23	<0.10	0.23	4.73	5.33	4.76	53.7
100 µg/mL	<0.10	<0.10	<0.10	<0.10	<0.10	0.7	1.62	0.89	9.01
10 µg/mL	<0.10	<0.10	<0.10	<0.10	<0.10	0.77	1.73	0.72	13
1 µg/mL	<0.10	<0.10	<0.10	<0.10	<0.10	1.62	1.88	1.6	21.8
0.1 µg/mL	<0.10	<0.10	<0.10	<0.10	0.24	4.73	5.37	4.59	45.9
0.01 µg/mL	0.18	<0.10	0.18	<0.10	0.22	4.95	5.32	4.64	55.7

Abbreviations: BM, buffalo milk; PBS, phosphate-buffered saline.
